# Evaluation of in vitro digestibility of *Aspergillus oryzae* fungal biomass grown on organic residue derived-VFAs as a promising ruminant feed supplement

**DOI:** 10.1186/s40104-023-00922-4

**Published:** 2023-10-01

**Authors:** Clarisse Uwineza, Mohammadali Bouzarjomehr, Milad Parchami, Taner Sar, Mohammad J. Taherzadeh, Amir Mahboubi

**Affiliations:** Swedish Centre for Resource Recovery, University of Borås, 50190 Borås, Sweden

**Keywords:** *Aspergillus oryzae*, Fungal biomass, In vitro dry matter digestibility, Ruminant feed, Volatile fatty acids

## Abstract

**Background:**

As demand for high quality animal feed continues to raise, it becomes increasingly important to minimize the environmental impact of feed production. An appealing sustainable approach to provide feed fractions is to use organic residues from agro-food industry. In this regard, volatile fatty acids (VFAs) such as acetic, propionic and butyric acids, derived from bioconversion of organic residues can be used as precursors for production of microbial protein with ruminant feed inclusion potential. This study aims to investigate the in vitro digestibility of the *Aspergillus oryzae* edible fungal biomass cultivated on VFAs-derived from anaerobic digestion of residues. The produced fungal protein biomass, along with hay clover silage and rapeseed meal were subjected to various in vitro assays using two-stage Tilley and Terry (TT), gas, and bag methods to evaluate and compare its digestibility for application in ruminant feed.

**Results:**

The produced fungal biomass contained a higher crude protein (CP) (41%–49%) and rather similar neutral detergent fiber (NDF) (41%–56%) compared to rapeseed meal. The rumen in vitro dry matter digestibility (IVDMD) of the fungal biomass in the TT method ranged from 82% to 88% (statistically similar to that of the gas method (72% to 85%)). The IVDMD of fungal biomass were up to 26% and 40% greater than that of hay clover silage and rapeseed meal, respectively. The type of substrate and bag method had pronounced effect on the fermentation products (ammonium-N (NH_4_^+^-N), total gas and VFAs). Fungal biomass digestion resulted in the highest release of NH_4_^+^-N (340–540 mg/L) and the ratio of acetate to propionate ratio (3.5) among subjected substrates.

**Conclusion:**

The results indicate that gas method can be used as a reliable predictor for IVDMD as well as fermentation products. Furthermore, the high IVDMD and fermentation product observed for *Aspergillus oryzae* fungal biomass digestion, suggest that the supplementation of fungal biomass will contribute to improving the rumen digestion by providing necessary nitrogen and energy to the ruminant and microbiota.

## Introduction

Livestock production systems are overstretched due to the continued growth of the world's population, accompanied by the challenges of high demand for animal-based protein products [1]. The supply of animal-based products, particularly from the ruminant sector, is associated with the increasing provision of ruminant feed, including feed-grade protein, through conventional protein sources. Until now, high-quality plant protein meals and co-products of oil extraction, especially from soybean, rapeseed, linseed, peanut, and sunflower seed, have been the most popular protein sources for ruminant nutrition [2].

Using high quality plant based protein meals in livestock productions raises concerns such as competition with human food, and the impact on the land use [3]. Therefore, ensuring sustainability and addressing ecological constraints is crucial when providing alternative protein supplements sources for ruminants. The quest for alternative protein supplement for animal feed has been ongoing for decades, focusing on obtaining them from the bioconversion of low-value residues to meet both sustainability and economic feasibility in livestock production. In this context, single-cell proteins (SCPs) have emerged as the most investigated sustainable alternative protein supplement.

SCPs derived from bacteria, microalgae, and fungi including yeast and filamentous fungi, primarily consist of protein along with carbohydrates, fats, minerals, nucleic acids and vitamins [4–6]. The nutritional quality of filamentous fungal biomass has attracted research and industrial interest in human food ingredient production. Certain filamentous fungal species, like *Aspergillus* spp., *Rhizopus* spp., *Neurospora* spp., and *Fusarium* spp., are considered safe for food and feed applications due to their use in East and Southeast Asian fermented foods, (e.g., oncom, koji and miso) and beverages (e.g., sake, shoyu, and vinegar) [5, 6]. Filamentous fungi, such as *Aspergillus oryzae (A. oryzae),* have shown the ability to grow well on low-value organic residues and yielding valuable products such as enzymes, alcohols, organic acids, and protein-rich biomass, as reported by numerous research studies [7–9]. Fungal biomass can contain approximately 60% crude protein (CP) and possess an amino acids and fatty acids profile similar to traditional ruminant feed protein sources like soybean meal, rapeseed meal and fish meal [10].

Filamentous fungi with their robust enzymatic machinery, can be sustainably cultivated on diverse organic residues, but the complexity and heterogeneity of organic waste streams hinder their bioconversion efficiency and affect the nutritional composition of the final fungal biomass. Anaerobic digestion (AD) is an effective bioconversion process that converts the mixed complex organic matter into volatile fatty acids (VFAs) during the stages of acidogenesis and acetogenesis. These VFAs, composed mainly of acetic, propionic and butyric acids and other valuable nutrients, can be valuable resources for producing protein-edible fungal biomass without additional chemical supplementation [11]. Previous studies successfully used VFAs from anaerobic digestion of animal manure and food waste to produce *A. oryzae* fungal biomass, exhibiting similar protein content to familiar animal feed protein sources like soybean and rapeseed meals [9].

However, replacing conventional ruminant feed fractions with fungal biomass requires considering its digestibility beyond protein content and quality. Although *A. oryzae* fermentation culture has been widely studied as microbial feed additives with prebiotic and probiotic effects in ruminant production [12–18], there is a lack of thorough digestibility studies on *A.oryzae* biomass as an alternative feed supplement for ruminant. In this regard, in vitro digestibility methods such as two stage Tilley and Terry (TT) [19] and gas production methods [20] are employed to assess the nutritional value of ruminant feed fractions. To the authors’ knowledge, no comprehensive in vitro digestibility study has been conducted on *A. oryzae* biomass grown on organic residue derived-VFAs, leaving a gap in understanding its quality and extent of rumen digestibility. Therefore, the hypothesis is that a comprehensive digestibility study will demonstrate the potential of *A.oryzae* biomass as a reliable and sustainable feed supplement in ruminant nutrition, addressing the existing gap in knowledge regarding its rumen digestibility and overall suitability as a feed supplement.

This study aims at initially cultivating *A. oryzae* biomass on different waste-derived volatile fatty acids (food waste, and potato protein liquor) and synthetic glucose media, followed by the evaluation of in vitro digestion behavior of the fungal biomass in comparison to hay clover silage and rapeseed meal. To assess all aspects of fungal biomass digestion from dry matter digestibility to the extent of gas and VFAs formation compared to common feed fractions, three standards in vitro digestibility methods of TT, gas method, and bag methods were applied, and digestibility results obtained were validated against one another.

## Materials and methods

### Microorganism

*Aspergillus oryzae* var. *oryzae* CBS 819.72 (Centraalnureau Voor Schimmelcultures, Utrecht, and The Netherlands) was used as the fungal strain throughout this study. The fungus was grown on Potato Dextrose Agar (PDA) plates containing 4 g/L potato infusion, 20 g/L *D*-glucose, and 15 g/L agar. First, the spore solution of the fungal strain was spread with an L-shape disposable plastic spreader on PDA plates and then cultivated in an incubator for 72 h at 30 °C. After the incubation, the plates were stored at 4 °C until use.

### Fungal biomass cultivation

The fungus *A. oryzae* was cultivated in 4.5-L bench-top bubble column bioreactors (Belach Bioteknik AB, Stockholm, Sweden). *A. oryzae* fungal cultivations were performed by using different substrates of synthetic glucose medium, and VFA effluents from the acidogenic fermentation of potato protein liquor (PPL) and food waste plus chicken manure (FWCKM) [21]. The bioreactors and substrates were autoclaved at 121 °C for 20 min, before inoculation. After the sterilization, 2.7 L of each substrate were added into the bioreactors and reactors were inoculated with 20 mL/L of spore suspension (1.69 ± 0.19 × 10^7^ spores/mL) followed by cultivation at 35 °C. The initial pH was adjusted to 6.2 using 2 mmol/L NaOH. During the cultivation, the bioreactors were continuously aerated at an aeration rate of 0.5 vvm (volume of air per volume of medium per minute). After 48 h, the produced fungal biomass was harvested and washed with tap water, then oven-dried at 70 °C for 24 h. The composition of VFA effluents used for producing fungal biomass is presented in Table 1.

**Table 1:** Characterization of PPL and FWCKM-derived VFA effluents and glucose media used as substrates for *A. oryzae* fungal biomass cultivation

	**Parameter**	**PPL**	**FWCKM**
VFAs effluents	pH	7.15	6.07
	tCOD, g/L	13.60 ± 0.33	12.80 ± 1.13
	NH_4_^+^-N, mg/L	565 ± 19.14	1320 ± 56.57
	Acetic acid, g/L	5.27 ± 0.15	3.45 ± 0.10
	Iso-butyric acid, g/L	0.35 ± 0.01	0.37 ± 0.01
	Butyric acid, g/L	0.62 ± 0.01	1.29 ± 0.04
	Iso-valeric acid, g/L	0.17 ± 0.01	0.24 ± 0.01
	Propionic acid, g/L	0.99 ± 0.02	0.83 ± 0.02
	Valeric acid, g/L	0.02 ± 0.00	0.08 ± 0.00
	Total VFA, g/L	7.43 ± 0.21	6.24 ± 0.16
	Na, mg/L	381.50 ± 6.36	829.50 ± 16.26
	K, mg/L	4,396.00 ± 33.94	1,019.50 ± 13.44
	Ca, mg/L	146.50 ± 3.53	49.72 ± 0.07
	Fe, mg/L	1.00 ± 0.00	1.00 ± 0.00
	Mg, mg/L	94.00 ± 0.00	34.00 ± 1.41
Synthetic glucose medium	Yeast extract, g/L	5.00 ± 0.01	
	*D*-glucose, g/L	30.00 ± 0.01	

*tCOD* Total oxygen demand, *NH*_*4*_^+^*-N* Ammonium-N, *VFAs* Volatile fatty acids, *FWCKM* VFAs effluents obtained from anaerobic digestion of food waste and chicken manure, *PPL* VFAs effluents obtained from anaerobic digestion of fermented potato protein liquor, *Na* Sodium, *K* Potassium, *Ca* Calcium, *Fe* Iron, *Mg* Magnesium, *TN* Total nitrogen. The sign ± stand for the standards deviation on two direction (positive and negative)

### Materials and rumen fluid

In the next step of the experiment, *A. oryzae* fungal biomass grown on glucose and VFAs, hay clover silage and rapeseed meal was investigated for rumen in vitro digestibility behavior. The rapeseed meal (EXPRO) (heat-treated rapeseed under pressure to increase the proportion of by-pass protein) and hay clover silage were supplied by Hushållningssällskapet Sjuhärad (Rådde gård, Länghem, Sweden). Prior to use, all materials were dried at 60 °C for 24 h and then milled, sized, and sieved to 1 mm using Pulverisette 14 (Fritsch, Germany). Briefly, 0.75 g of each material (three different fungal biomass, rapeseed meal and hay clover silage) were weighted and incubated with a mixture of rumen fluid and buffers (ratio of 1:4). All in vitro assays were triplicated.

The ruminal fluid was provided by the Swedish Livestock Research Centre (SLU Lövsta lantbruksforskning, Uppsala, Sweden), collected from three non-lactating cows with access to a diet consisting of hay plus concentrate (Lantmännen Feed) for their morning and evening meal, and straw plus minerals for their mid-day meal for one week. The rumen fluid was taken 2.5 h after the morning feeding from the rumen of each cow and collected in a prewarmed (39 °C) thermo-flask. Thereafter, the rumen fluid was sieved through three layers of cheesecloth while under constant CO_2_ purging before keeping at 38–39 °C until use [19].

The used buffer solution for all in vitro digestibility methods was prepared according to McDougall [22] based on the synthetic saliva formula (g/L) 0.57 KCl, 0.47 NaCl, 9.8 NaHCO_3_, 9.3 Na_2_HPO_4_·12H_2_O, 0.04 CaCl_2_ anhydrous and 0.06 MgCl_2_ anhydrous. The prepared buffer solution was saturated with CO_2_ until it become clear and kept in water bath at 39 °C prior to loading.

### In vitro digestibility

The in vitro assays were performed through three experimental methods using McDougall [22] and rumen fluid as follows:

- **Two stage in vitro dry matter digestibility: Tilly and Terry method**

In vitro dry matter digestibility (IVDMD) was analyzed according to Tilley and Terry [19] and modified using a 750-mg sample size and the addition of pepsin after 48 h. Briefly, 750 mg of each material (three different fungal biomass, rapeseed meal and silage) was weighted in a 125-mL screw cap glass bottle with a hole and septum containing rumen fluid and buffer solution at a ratio of 1:4 (15:60 mL). After sample preparation, each of the bottles with the material plus 3 bottles of blanks (only rumen and buffer) were purged with CO_2_ for 2 min and placed in a pre-warmed water bath at 39 °C shaking at 100 r/min for 48 h. In addition, at 6 h and 24 h of the incubation process, the pH of all conditions was adjusted to 6.9 using 1 mol/L Na_2_CO_3_ (sodium carbonate (Sigma-Aldrich and Merck, Germany). After 48 h, the media were acidified to a pH of 1.5 using 2.2 mol/L HCl (Sigma-Aldrich and Merck, Germany) [19]. After acidification, 2% pepsin (≥ 3.200 unit/mg protein) was added to each bottle and placed in a water bath for 48 h. In the end, the residuals were centrifuged at 1,500 × *g* for 10 min followed by 3 times washing. After centrifugation, the residuals were dried in the oven at 60 ℃ for 24 h until constant weight and used to calculate apparent IVDMD. For all methods, IVDMD% of remaining biomass after the digestion was evaluated based on dry matter disappearance (g/kg) [19]:

$$IVDMD \% =\left(\left({DM}_{Initial}-\left({DM}_{Residual}-Blank\right)\right)\div {DM}_{Initial}\right)\times 100$$

where IVDMD % is the percentage of in vitro dry matter digestibility, DM_initial_ is the initial dry matter of sample loaded to the process (g), DM_Residual_ is the amount of DM of the residual biomass after digestion process (g), Blank is the amount of dry matter of the biomass in blank solutions (g).

b. **In vitro gas production method**

The in vitro gas production was performed for 48 h by monitoring fermentation products, including the concentration of total accumulated gas, CO_2_, H_2_, CH_4_, VFAs, ammonia nitrogen and the collection of residuals used to determine the apparent IVDMD. Each batch assay was triplicated. Simply, 750 mg DM of each sample material were mixed with 15 mL of rumen fluid and 60 mL buffer solution (1:4 ratio) into a 125-mL screw cup glass bottle having septa on the cap; in total, 15 bottles plus three bottles of the blank were used. Before incubation, all 18 bottles were sealed, purged with CO_2_ for 2 min and placed into a pre-warmed water bath at 39 ºC shaking at 100 r/min for 48 h. The gas samples were taken manually with a syringe at 3, 6, 9, 12, 24, 32, and 48 h, while 2 mL sample aliquots were taken at 6, 12, 24, and 48 h for VFAs and ammonia nitrogen analysis. After 48 h, the residuals were centrifuged at 1,500 × *g* for 10 min and washed thrice. The residuals were collected, filtered, and oven-dried until constant weight to determine apparent IVDMD.

c. **In vitro bag method**

The process of in vitro bag method was performed by modifying a method developed by Goeser and Combs [23]. For the bag method, 750 mg of each material was loaded in a pre-weighed Ankom F-57 polyester/polyethylene bag with 5 cm × 5.5 cm and a pore size of 25 µm (ANKOM Technology, Macedon, USA). The bags with the material plus empty bags were heat sealed and placed into 125-mL bottles filled with 10 mL rumen fluid and 60 mL of buffer solution. All sample materials were prepared in triplicates, including blanks. From this point, the same procedure as for the gas method was applied. At the end of the experiment, the bags with the residuals inside were washed with deionized (Milli-Q) water three times till the liquid became clear and dried at 60 ºC for 24 h until constant weight for IVDMD calculation [23].

### Analytical methods

The supplied hay clover silage and rapeseed meal complete analysis was performed using NIR methods at EUNLWA2 (Eurofins Agro Testing Sweden AB, Kristianstad, Sweden). The dried and sized hay clover silage and rapeseed meal, together with fungal biomass, were further analyzed, and the results are presented in Table 1. Dry matter (DM) was evaluated by drying 10 g of material in an oven at 105 ºC for 24 h until constant weight. The dry matter residuals were incinerated at 550 ºC for 12 h to determine organic matter (OM) and ash. Crude protein was obtained according to AOAC official method 954.01 [24] by multiplying a factor of 6.25 with total Kjeldahl nitrogen (TKN). The TKN was analyzed according Kjeldahl method using an InKjel digester and a behrostest S1 distiller (Behr Labor-Technik, Germany). The method procedure start with the addition on 20 mL of 98% H_2_SO_4_ (Sigma-Aldrich and Merck, Germany), KT1 and antifoam tablets (Tompson & Capper Ltd, Runcorn, UL) to the pre-weighed 0.5 g dried sample prior to digestion 100 min. After digestion, the digested material are subjected to the distillation unit connected to 50 mL of 4% boric acid (H_3_BO_4_) for vapor collection. During the last step of titration, 0.1 mol/L HCl (Sigma-Aldrich and Merck, Germany) is used to reach the pH of 4.6, and the volume is collected and used to calculate TKN [25]. The mineral ion content of the medium (sodium, potassium, calcium, iron, and magnesium) was determined using Microwave Plasma-Atomic Emission (MP-AES 4200, Agilent Technologies, Santa Clara, CA, USA). Alkali insoluble materials (AIM) was analyzed according to the method by Zamani et al. [26]. Crude fat content was determined using petroleum ether extraction by Soxtec extraction analyser (ST243 Soxtec™, FOSS Analytical Co., Ltd., Suzhou, China). After the extraction, the amount of fat was calculated based on the evaporated organic solvent. The fiber content were extracted using ANKOM 200 Fiber Analyzer. The samples were sealed with heat sealer (HS or His, ANKOM Technology, Macedon, USA) in the filter bag (F57 and F58, ANKOM Technology, Macedon, USA). The ready and weighted bags with samples were then placed in a digesting instrument for extraction (ANKOM 2000 agitating at 65 r/min, ANKOM Technology, Macedon, USA). During digestion different solvent were used based on the analysis. Crude fiber (CF), neutral detergent fiber (NDF), and acid detergent fiber (ADF) were determined according to the methods procedures found at Ankom [27].

The concentration of VFAs in the substrates used for fungal cultivation and the changes in the VFAs content of digestion batches were analyzed using Gas Chromatography (GC) instrument (Clarus 550; Perkin—Elmer, Shelton, CT, USA), which was equipped with a flame ionized detector (FID) and a capillary column (Elite-Wax ETR, 30 m × 0.32 mm × 1.00 µm, Perkin—Elmer, Shelton, CT, USA). For GC-FID analysis, nitrogen gas was used as carrier gas at a pressure of 1.38 bar and a 2 mL/min flow rate. The injection and detection temperatures were 250 ºC. Prior to VFA analysis, samples were centrifuged (1,200 × *g* for 5 min), 1 mL of supernatant was transferred into 1.5-mL Eppendorf tubes, mixed with 200 µL acid mix solution (containing 25% (v/v) ortho-phosphoric acid and 25% (v/v) formic acid at a ratio of 3:1), vortexed, centrifuged (1,200 × *g* for 5 min) and filtered through 0.2-µm syringe filter. Then, 250 µL of supernatant was mixed with 250 µL butanol (1 g/L) (internal standard) and 500 µL milli-Q water.

The produced gas composition of CO_2_, CH_4_, and H_2_ was measured semi-automatically using a GC instrument (Clarus 550; Perkin—Elmer, Shelton, CT, USA). The gas sample volume of 0.25 µL was injected in a packed column (CarbonxenTM 100, 6' × 1.8' OD, 60/80 mesh, Suplco, Shelton, CT, USA), set at 200 ºC and nitrogen gas as carrier gas flowing at a rate of 30 mL/min. Once injected, the gas composition was detected with a thermal conductivity detector (TCD) [28]. The gas volume was recorded manually over both high and low gas pressure, normalized to the standard atmospheric pressure and normal temperature of 0 ºC and 20 ºC and used to determine the cumulative gas volume and total gas produced at each sampling time [28].

Total chemical oxygen demand (COD) and ammonium-N (NH_4_^+^-N) content of the media was measured using Nanocolor® COD 15000 and Nanocolor® Ammonium (Duren, Germany) analysis kits using a Nanocolor 500D photometer (MACHERY-NAGEL GmbH & Co. KG, Germany), respectively.

### Statistical analysis

All experiments were conducted in triplicates and the acquired results were statistically analyzed using Minitab 21®. Analysis of variance (ANOVA) was performed on the result using one way ANOVA, where significant difference in results obtained were defined at *P* < 0.05 within 95% confidence intervals followed by pairwise comparisons according to turkey's test.

## Results and discussion

Filamentous fungal protein is a sustainable alternative protein source that can be produced through the bioconversion of low-value residues. In the current study, *A. oryzae* fungal biomass cultivated on VFA effluents derived from anaerobic digestion of organic residues were characterized and compared with hay clover silage and rapeseed meal. These components were then subjected to the rumen in vitro digestion tests. The extent and rate of digestibility, fermentation products (gas, VFAs, and ammonia nitrogen), and dry matter disappearance were determined using Tilly and Terry, gas, and nylon bag methods.

### Composition of *Aspergillus oryzae* biomass

The produced fungal biomass, cultivated on VFAs from anaerobic digestion of organic residues and glucose-based media, is characterized and compared with silage and rapeseed meal commonly used for ruminant feeding in Table 2. The crude protein content of the fungal biomass varied between 41% and 49%. Comparing the compositions presented in Table 2, fungal biomass contained greater crude protein than silage (17.9% CP) and rapeseed meal (39% CP) (*P* < 0.001). Alkaline insoluble material (AIM) of all produced fungal biomass was 31%–32%, while lower levels of around 28% and 18% were detected for rapeseed meal and silage, respectively (*P* < 0.001). The fungal biomass's ash (inorganics) content is about 6.2% from synthetic glucose medium, while it is between 10%–15% from VFA-containing effluent. The ash levels of rapeseed meal and silage were between 5% and 7%. All biomass’ CF, NDF, and ADF were analyzed (Table 2). In this regard, CF levels for FWCKM and PPL-based fungal biomass were approximately 41%, while for the fungi cultivated on a synthetic glucose media, the CF content was about 10% higher (*P* = 0.003). The NDF content of the fungal biomass ranged from approximately 41% to 56%, and it was greater than NDF in rapeseed meal and silage of 37% and 38%, respectively (*P* < 0.001). *A. oryzae* grown on glucose had the highest average amount of ADF, of 55%, than the ADF percentage for *A. oryzae* grown on VFAs from PPL (AO_VFAs PPL) and food waste and chicken manure (AO_VFAs FWCKM) of 43% and 41%, respectively (*P* = 0.010).

**Table 2:** The characteristics of the produced *A. oryzae* fungal biomass compared to hay clover silage and rapeseed meal

Item	**AO_ VFAs FWCKM**	**AO_ VFAs PPL**	**AO_ Glu.**	**Rapeseed meal**	**Hay clover silage**	**SEM**	***P*-value**		
							**Fungal biomass vs. rapeseed meal**	**Fungal biomass vs. silage**	**Rapeseed meal vs. silage**
OM, g/kg DM	842.11^e^	891.05^d^	938.03^b^	930.99^c^	946.76^a^	1.59	0.001	0.001	< 0.001
AIM, % DM	31.38^a^	31.52^a^	31.14^a^	28.19^b^	17.92^c^	0.40	< 0.001	< 0.001	< 0.001
Ash, % DM	15.79^a^	10.89^b^	6.19^ cd^	6.91^c^	5.32^d^	0.54	0.005	0.005	0.044
Fat, % DM	1.56^e^	1.65^d^	2.35^c^	4.20^a^	3.50^b^	0.03	< 0.001	< 0.001	< 0.001
CP, % DM	49.65^a^	41.05^b^	41.73^c^	39.08^d^	18.03^e^	0.02	< 0.001	< 0.001	< 0.001
CF, % DM	39.02^c^	42.2^b^	51.35^a^	28.71^e^	32.32^d^	0.77	< 0.001	< 0.001	0.001
ADF, % DM	39.92^c^	42.83^b^	55.61^a^	40.34^c^	22.00^d^	0.39	< 0.001	< 0.001	< 0.001
NDF, % DM	41.78^c^	43.66^b^	56.89^a^	37.6^d^	38.10^d^	0.52	< 0.001	< 0.001	0.700

*DM* Dry matter, *OM* Organic matter, *AIM* Alkali insoluble material, *CP* Crude protein, *CF* Crude fiber, *ADF* Acid detergent fiber, *NDF* Neutral detergent fiber, *VFAs* Volatile fatty acids, AO_VFAs FWCKM: *A. oryzae* biomass grown on VFAs effluents from food waste and chicken manure; AO_VFAs PPL: *A. oryzae* biomass grown on VFAs effluents from fermented potato protein liquor; AO_Glu.: *A. oryzae* biomass grown on glucose. Values with different superscript letter in a raw are significantly different at 5% turkey test

The obtained fungal biomass protein content results were found to be comparable with previously reported crude protein contents of *A. oryzae* (37%–41%) and strains cultivated on short-chain carboxylic acids [9]. Rapeseed meal is abundantly used as feed ingredients for various animal species such as poultry, pigs, cattle and fish [29]. Protein is an essential ingredient in ruminant feeding. There exist two specific types of protein in ruminant feed. The available protein that provides the source of N to rumen microbes is known as rumen degradable protein (RDP), and the protein not degraded by rumen microbes and available to the animal for lactation and tissue growth is known as rumen undegradable protein (RUP). RDP play a crucial role in boosting microbial activity, rumen microorganism growth and mainly microbial protein synthesis, which contribute to microbial protein flow to the intestine to be absorbed and used by the animal. RUP is the protein not degraded in the rumen; it is absorbed and used by an animal as metabolizable protein passes through its digestive tract. Estimating RDP and RUP is the central theme of determining ruminant feed protein quality. The feed protein quality and availability of nutrients influence the animal's performance. In general, hay clover silage provides essential fermentable protein and carbohydrates for the rumen, while heat-treated rapeseed meal or other processed protein feedstuff provides a quality amount of undegradable rumen protein absorbed in the intestine [30]. The quality of fungal protein is discussed in the following sections based on ammonium release after ruminal digestion.

Alkali insoluble fraction generally represents fungal cell wall components such as β-glucan, chitin, etc. [31]. Since these components are bioactive compounds, they can regulate immunity, increase antioxidant capacity, and reduce inflammatory response when used as animal feed [32]. Due to the higher content of elemental minerals in VFA-based effluents, it is hypothesized that the fungi potentially absorbed a considerable amount of these elements from the media. Minerals are essential for the health and well-being of animals. In Karimi et al. [33] analysis, the *A. oryzae* fungal biomass grown on vinasse contained a substantial amount of Ca, P and K that was higher than that of soybean meal and fish meal. According to National Research Council [30], estimates of the required minerals in ruminant feed vary depending on the animal's level of productivity and physiological state. For instance, 0.30%–0.40% Ca, 0.10%–0.26% P and 0.5%–0.7% K for beef cattle and growing animals and 0.43%–0.60% Ca, 0.31%–0.40% P and 0.80%–1.2% K for lactating animals (% based on diet dry matter). Therefore, the capability of fungi to take up essential minerals from low-value substrates can play an important role in ruminant nutrition.

The percentage of CF for silage was about 32%, similar to that previously reported [34]. It is well known that NDF covers all cellulose, hemicellulose and lignin components in plant cells. ADF includes only cellulose and lignin, whilst CF does not quantitatively represent a hemicellulose and lignin recovery [30]. For this reason, NDF is commonly used to indicate the structural carbohydrate content in ruminant feed. Although NDF is a vital source of fermentable structural carbohydrates for ruminants and a significant energy source for daily cattle, higher NDF content does not necessarily mean higher digestibility. The extent of NDF degradability indicates feed quality, as diets containing highly degradable fiber result in higher digestible energy and forage intake [35]. Rapeseed meal is one of the most widely used protein sources for ruminant feed. However, rapeseed meals contain high NDF, mainly from hulls, resulting in a lower digestible energy content than other protein sources such as soybean meal [36]. The NDF in silage mostly comes from plant cell walls and is reported to be easily digestible in rumen [37]. On the other hand, the NDF content in fungal biomass can be considered a non-forage fiber source that can be compared with industrial byproducts such as distiller grains, which are mainly rich in digestible NDF [38, 39]. Therefore, it can be stated that the filamentous fungal biomass is sufficient in crude nutrients, suitable for ruminal fermentation and alternative nutrient additive.

### In vitro dry matter digestibility (IVDMD)

**Fig. 1:**
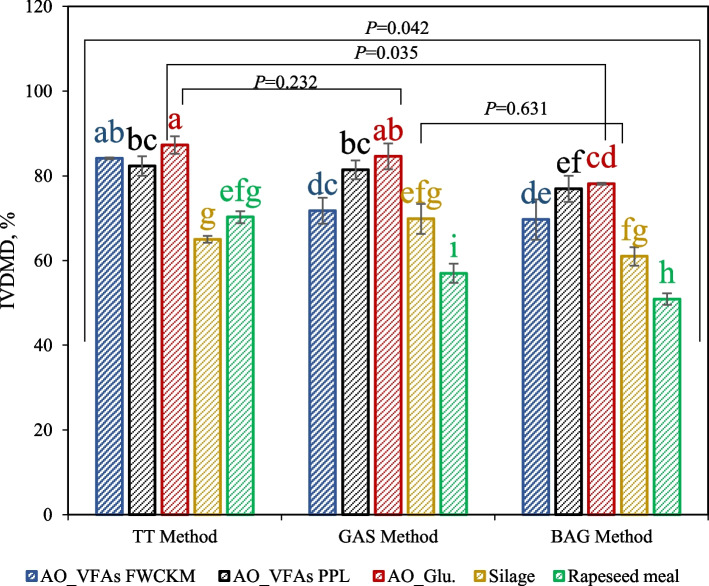
The determined IVDMD of the *A. oryzae* fungal biomass, hay clover silage and rapeseed meal from TT, gas and bag methods digestion assays. Values with different letter on columns are significantly different at 5% turkey test

The residues of the fungal biomass and references feed samples after digestion with the findings are illustrated in Fig. 1. The rumen IVDMD of the fungal biomass in TT, gas and bag methods ranged from 69.69% to 87.29%, which were on average 3%–26% and 15%–40% higher than those of silage (*P* = 0.034) and rapeseed meal (*P* < 0.001), respectively. It was observed that the type of material had more effect on the deviation of dry matter disappearance obtained within each method. For example, the IVDMD of fungal biomass varied widely, with fungal biomass grown on glucose being the most digestible in all methods compared to fungal biomass based on VFAs. Fungal biomass from VFAs had a higher ash content (mineral content) than glucose-based biomass, indicating of lower digestibility compared to glucose-based biomass (Table 2).

Filamentous fungal biomass produced from VFAs effluents and other organic-rich streams could assimilate macro-minerals, particularly calcium, potassium, phosphorus, iron, sodium, and magnesium [9, 33]. As such, they can also be potential sources of macro-minerals in ruminant feed. Macro-minerals have an essential role in the diet of ruminants. They are mainly absorbed in the intestine and used by the animal for skeletal tissues and bone formation, acid–base balance and generally improve the animal's performance [30]. Although the obtained IVDMD of 50.90%–70.25% and 61.00%–69.84% for rapeseed meal and silage, respectively, was lower than that of fungal biomass (*P* = 0.000), these results complied with the other studies reporting rapeseed meal and silage in vitro digestibility [40]. The type of pretreatment on rapeseed meal can be one of the main reasons for the alternation in ruminal digestion results [41, 42].

According to the obtained results, when the material was placed in the bag, the difference in dry matter disappearances of fungal biomass, hay clover silage, and rapeseed meal was pronounced compared to the dry matter digestibility obtained in TT and gas methods. The IVDMD of fungal biomass obtained within TT methods (84%–88%) showed a significant difference with the bag method (69%–78%) (*P* = 0.020) but was rather similar to that obtained via the gas method (72%–85%) (*P* = 0.232). The IVDMD of silage and rapeseed meal observed in TT and bag methods compared well with other studies [40, 43]. However, based on previous studies, the generally lower digestibility values obtained for the bag method compared to other approaches could be due to the sample-to-bag size ratio [44], the presence of surfactant on bags, the sample size to a surface ratio [44, 45], and organic matter accessibility within samples in the bag [45]. Regarding the sample size to bag size ratio, it has been reported that increasing the ratio generally leads to a decrease in sample disappearance. This relationship of inhibiting dry matter digestion is associated with inadequate mixing, affecting fermentation rates. Therefore, the recommended ratio of a sample size to bag size is claimed to be between 10 and 20 mg/cm^2^ [46]. The ratio in this study was 13 mg/cm^2^. The presence of surfactant could also contribute to the low DM disappearance observed in the bag method. Adesogan [44] reported a similar conclusion when using an unwashed Ankom F-57 bags and an acetone pre-washed bag to remove the surfactants. They noticed that the unwashed bag underestimated the silage digestibility and recommended including the acetone pre-washed step. Regarding the availability of organic matter for microorganisms, Li et al. [47] reported that rumen ciliates cannot pass through Ankom F-75 bag with pore size of 25 µm for organic matter degradation. However, it is important to emphasize that rumen ciliates possess a wide range of carbohydrates-active enzymes capable of degrading microbial carbohydrates.

### Changes in ammonium-N content during in vitro digestion

**Fig. 2:**
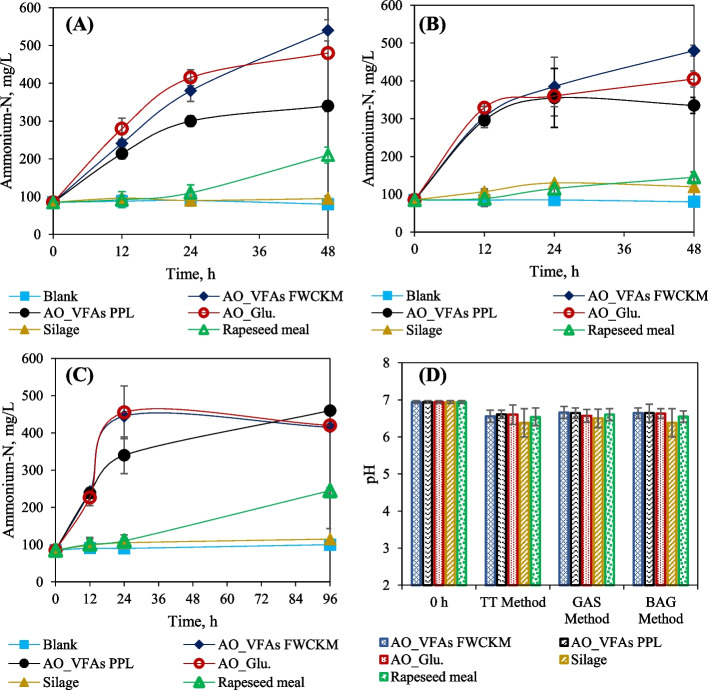
The recorded, ammonium-N concentrations and pH from in vitro digestion of *A. oryzae* fungal biomass, hay clover silage and rapeseed meal (**A**) Ammonium-N concentrations from gas method (**B**) Ammonium-N concentrations bag method, (**C**) Ammonium-N concentrations from TT method, and (**D**) Change in pH at 48 h in all methods

The differences in the DM digestibility recorded for fungal biomass, hay clover silage and rapeseed meal ingredients are also reflected in the ammonium-N released from CP hydrolysis. Ammonia-N, amino acids, and peptides are potential products during the ruminal digestion of rumen-degradable proteins. Ammonia-N released during the ruminal degradation of nitrogenous compounds such as amino acids, plays a significant role in ruminal microbial growth and presents the degree of protein availability and accessibility [48]. In this study, there was no significant difference in ammonium-N release by gas, bag and TT method (*P* = 0.928), (Table 3) and (Fig. 2A–C). In the gas method, the ammonium-N released from the fungal biomass of *A. oryzae* increased from 85 mg/L to an average of 365 mg/L, 4–5 times at a rate of 9–14 mg/L/h in just 24 h. After 48 h, the highest release was from the fungal biomass grown on glucose (415 mg/L), was up to 5 and 3.5 times higher than silage (90 mg/L) and rapeseed meal (110 mg/L), respectively (Fig. 2A). The high rate of ammonium-N release was highly related to the IVDMD and could be attributed to the higher CP and RDP in fungi compared to silage and rapeseed meal. This high ammonium-N release in 24 h could be explained by the rapid degradation of proteins, mainly related to the combination of proteolytic activities of bacteria, protozoa, and fungi. The liberated ammonia-N and free amino acids become the primary source of N for microbial growth. Although bacteria have been extensively known to have high proteolytic activities, Karimizadeh et al. [49] have also shown that the presence of protozoa enhances protein digestion, mainly if the dietary protein is slowly degradable. This gives rise to an increase in the rate of ammonium-N production between 4.16 and 5.62 mg/L/h within 24 to 48 h for rapeseed meal in gas and TT methods, respectively, while there were no significant changes with silage (Fig. 2A and C**).**

**Table 3:** The IVDMD, and fermentation quality of the tested *A. oryzae* fungal biomass, silage and rapeseed meal ingredients, and the comparisons of the methods at 48h

**Ingredients**	**GAS**					**BAG**					**TT**					**SEM**	***P*** **(GAS vs. BAG)**
	AO_ VFAs FWCKM	AO_ VFAs PPL	AO_ Glu.	Hay clover silage	Rapeseed meal	AO_ VFAs FWCKM	AO_ VFAs PPL	AO_ Glu.	Hay clover silage	Rapeseed meal	AO_ VFAs FWCKM	AO_VFAs PPL	AO_Glu.	Hay clover silage	Rapeseed meal		
IVDMD, %	72.66^de^	81.44^bc^	85.94^ab^	68.80^efg^	50.9^i^	69.69^de^	71.51^ef^	78.16^cd^	66.64^fg^	57.02^h^	84.11^ab^	82.33^bc^	88.63^a^	65.02^g^	70.25^efg^	1.94	0.631
NH_4_^+^-N, mg/L	540^a^	340^e^	480^b^	95^i^	210^g^	480^b^	335^e^	405^d^	120^hi^	155^h^	415^d^	460^bc^	430^cd^	115^i^	255^f^	12.82	0.974
Total Gas, mL/g DM	43.81^b^	39.47^cd^	53.19^a^	37.52^d^	36.58^d^	26.34^e^	31.92^de^	42.43^c^	19.67^f^	35.86^d^						3.01	0.763
H_2_, mL/g DM	5.87^bc^	6.43^b^	5.95^bc^	9.02^b^	2.34^d^	5.38^bc^	5.69^bc^	4.70^c^	6.86^b^	2.05^d^						0.89	0.370
CO_2_, mL/g DM	34.18^a^	29.41^abc^	32.68^b^	23.34^de^	21.67^de^	15.28^f^	20.26^e^	28.96^bc^	12.79^f^	24.73^cd^						3.2	0.003
CH_4_, mL/g DM	6.23^d^	11.40^b^	14.56^a^	5.46^d^	12.58^b^	5.65^d^	5.96^d^	8.75^c^	0.22^e^	8.84^c^						0.93	0.188
TVFAs, mmol/L	79.13^d^	93.28^ab^	99.23^a^	84.49^bcd^	85.32^bcd^	76.97^d^	87.30^bc^	84.61^bcd^	82.15^cd^	77.10^d^						3.13	0.003
Acetic, mmol/L	50.00^de^	58.06^b^	65.50a	49.40^de^	49.40^de^	50.96^cd^	56.45^bc^	51.45^cd^	45.96^de^	44.13^e^						2.17	0.040
Butyric, mmol/L	18.92^d^	22.46^abcd^	20.41^cd^	27.17^ab^	25.21^abc^	22.13^bcd^	20.32^cd^	21.95^bcd^	28.63^a^	25.31^abc^						2.04	0.553
Propionic, mmol/L	9.15^cd^	12.46^abc^	13.32^ab^	15.25^a^	10.69^bcd^	3.88^e^	10.53^bcd^	11.20^bc^	7.55^d^	7.66^d^						1.21	< 0.001

There are the *P*-value from the comparison of all methods at 48h (Gas vs. TT vs. Bag on IVDMD (0.042) and NH_4_^+^-N (0.928); *P*-value from the comparison of Gas vs. TT methods on IVDMD (0.232) and NH4^+^-N (0.984), *P*-value from the comparison of Gas vs. Bag methods on IVDMD (0.035) and NH4^+^-N (0.921)

IVDMD: In vitro dry matter digestibility; DM: dry matter; NH4^+^-N: Ammonium-N; H_2_: Hydrogen; CO_2_: Carbon dioxide; CH_4_: Methane; VFAs: Volatile fatty acids; AO_VFAs FWCKM: *A. oryzae* biomass grown on VFAs effluents from food waste and chicken manure; AO_VFAs PPL: *A. oryzae* biomass grown on VFAs effluents from fermented potato protein liquor; AO_Glu.: *A. oryzae* biomass grown on glucose

Values with different superscript letter in a raw are significantly different at 5% turkey test

Moreover, after 48 h of digestion of the fungal biomass in the gas method, a total amount of ammonium-N of 340–540 mg/L was recorded, which is up to 6 and 3 times higher (*P* <0.001) than silage (95 mg/L) and rapeseed meal (210 mg/L), respectively (Fig. 2A). The increased ammonium-N after 24 h could also be due to the continuous deamination of amino acids and peptides. When the proteins are digested in the rumen, the peptides are released from the rumen degradable protein, which is later hydrolyzed into amino acids, some of which are deaminated into ammonia-N. However, Russell et al. [50] pointed out that in addition to degradable rumen protein degradation, rumen energy level and efficiency of microbial protein synthesis are the other factors that can affect the content and the use of ammonia-N. This theory would imply that when there is an energy limitation for bioconversion, there is a less direct use of ammonia-N for microbial protein synthesis or VFA production, resulting in an accumulation of ammonia-N [50]. Accordingly, the higher release of ammonium-N during the digestion of fungal biomass indicates its generation from degradable proteins in the rumen.

On the other hand, the higher ammonium-N concentration recorded as a result of fungal biomass digestion can be an indication that adding *A. oryzae* fungal biomass stimulates the protease activity for protein hydrolysis into amino acids and increased the deamination of amino acids into ammonia. Pilachai et al. [51] and Manoukian et al. [52] confirmed that supplementing highly degradable rumen protein could stimulated rumen fermentation efficiency. They concluded that the provided degradable rumen protein can supply the ammonia and amino acids necessary for efficient ruminal bacterial growth and increasing dry matter digestibility. Besides, Yoon and Stern [12] reported that the supplementation of *A. oryzae* fungal biomass as a microbial feed additive increases fiber degradation and microbial growth, especially cellulolytic and proteolytic bacteria, which are the main contributors to protein degradation in the rumen. This finding was consistent with that of Frumholtz et al. [53], who reported that the addition culture extract of *A. oryzae* improved ammonia concentration and viable bacterial and cellulolytic population resulting in the increased rumen and total tract digestibility of fibers fractions. On the other hand, ammonia deficiency disturbs carbohydrate degradation and microbial growth [54]. The lower CP of silage compared to rapeseed meal and fungal biomass content in silage was strongly associated with lower ammonium-N released during digestion. It appear that the silage likely contains highly soluble proteins that are degraded rapidly by the synthetic effect of microbial activity and plant protease activity into ammonia-N, as Zhang et al. [55] reported for alfalfa silage and stylo silage.

The ammonium-N released during digestibility tests did not have extreme effects on the pH of the medium in the in vitro digestibility assays (Fig. 2D). The pH ranged between 7.04 and 6.38, in the normal rumen pH ranged from 5.5 to 7.0 [56]. However, further research is needed to assess the levels of ammonium-N release and the dry matter digestibility of fungal biomass and its effect on the rumen microbiota and fermentation efficiency.

### Fermentation performance during in vitro digestion

**Fig. 3:**
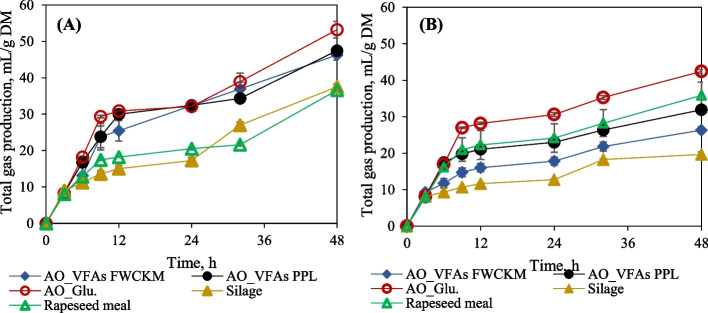
In vitro total gas production (mL/g DM) from *A. oryzae* fungal biomass hay clover silage and rapeseed meal in (**A**) gas and (**B**) bag methods

**Fig. 4:**
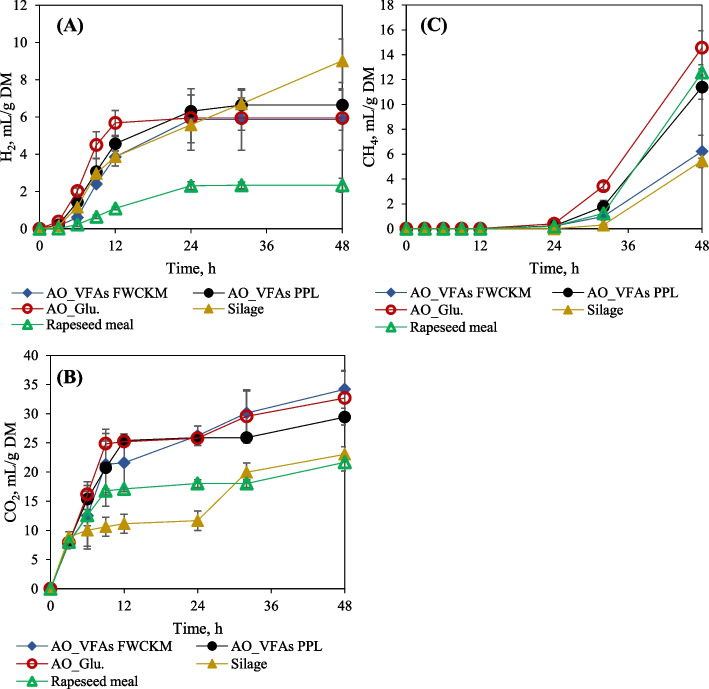
In vitro gas production and accumulation from *A. oryzae* fungal biomass hay clover silage and rapeseed meal in gas method (**A**) H2, (**B**) CO2, and (**C**) CH4 in mL/g DM

**Fig. 5:**
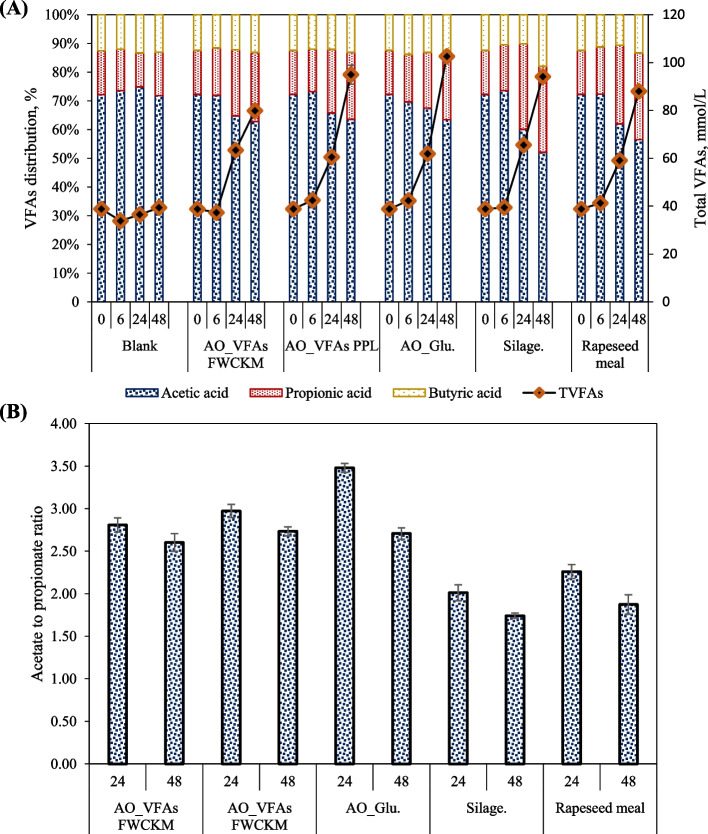
In vitro fermentation products from the digestion of *A. oryzae* fungal biomass, hay clover silage and rapeseed meal in gas method; (**A**) total VFAs, acetic acid, butyric acid and propionic acid and (**B**) acetate to propionate ratio

In general, the production of gas, VFAs and microbial growth are closely related to the digestion of substrates. In this study, the total accumulated gas, gas distribution (H_2_, CH_4_ and CO_2)_ and total VFAs and their distribution presented in Figs. 3, 4 and 5 were measured for all in vitro digestion methods at different sampling intervals according to the protocols. In general, the total gas produced from the gas method (Fig. 3A), where the input material was in direct contact with rumen microorganisms, was 1.1–2 times higher (*P* = 0.034) than the results of the bag method (Fig. 3B), with the highest recorded for AO_Glu. (53.19 mL/g DM) and the lowest for hay clover silage (19.67 mL/g DM). These differences could be attributed to the factors mentioned in previous sections, such as the presence of surfactant material that could clog the bag pores resulting in poor mass transfer and hence organic matter accessibility. A study by Cone et al. [57] suggested that gas production in nylon bags can be only related to the fermentation of non-water soluble components, where total gas production from the standard gas production method is a result of fermentation of the total sample, including soluble and non-soluble components and microbial turnover. For this reason, due to the higher gas produced in fungal biomass (42.43 mL/g DM) in bag method (*P* < 0.001), (Fig. 3B) comparing to that of hay clover silage (19.67 mL/g DM) and rapeseed meal (35.86 mL/g DM), it can be perceived that it is due to higher content of degradable non-water-soluble components (either both protein and fiber (NDF) in fungal biomass.

As mentioned, sample type directly affected the gas release apart from the method applied for the dry matter digestibility or the dry matter digestibility, sample type directly affected the gas release apart from the method applied. The total gas production (gas method) from fungal biomass that ranged from 39.37 to 53.19 mL/g DM was 1.53–2 times (*P* < 0.001) higher compared to hay clover silage 47.52 mL/g DM and rapeseed meal 36.58 mL/g DM** (**Fig. 3A). There was no significant difference (*P* = 0.988) in total gas produced from different waste-derived fungal biomass ranged between 47.45 and 53.19 mL/g DM. About 54%–63% of total gas was produced in the first 12 h at a rate of 2.14–2.50 mL/h from fungal biomass, 39% at a rate of 1.14 mL/h from hay clover silage and 49% at a rate of 1.48 mL/h from rapeseed meal, respectively. The higher gas production rate during the digestion of fungal biomass may indicates that the biomass provides a higher content of readily rumen degradable carbohydrates and CP, which are necessary for microbial growth. This is consistent with Cone et al. [58], who reported three stages of gas buildup during animal feed fermentation. They concluded that early gas production between 1 and 12 h is strongly related to soluble fractions, especially crude protein. The second stage, which lasts up to 30 h, indicates the fermentation of insoluble but degradable fractions, mainly starch and NDF. And finally, the thirst stage suggests the microbial population turnover, which could be detected after the depletion of the substrate. In general, in vitro fermentation of protein feed ingredients results in low gas production compared to carbohydrate-rich feedstuff, caused by the inhibition of high ammonia-N, as explained by Cone and van Gelder [59]. On the other hand, low gas production from heat-treated rapeseed meal can be due to the fact that it has been processed to resist ruminal fermentation. Even though fungal biomass contained higher fiber (NDF) content than hay clover silage and rapeseed meal, it had the lowest residues left after digestion. The produced gas in fungal biomass was highly linked to disappeared dry matter (Fig. 1). According to Van Soest et al. [35], the low amount of residues left after digestion is directly related to the gas produced and the apparent IVDMD. Evidently, the cell wall fraction of microbial biomass does not contain highly recalcitrant lignocellulosic cell wall components such as lignin present in silage, signaling its higher digestibility [60].

The produced gas distribution is presented in Fig. 4 as volume per added dry matter of fungal biomass, hay clover silage and rapeseed meal. As mentioned above, more than 50% of gas was produced in the first 12 h of digestion. As presented in Fig. 4A, the fungal biomass produced from glucose released the highest H_2_ of 5.56 mL/g DM after 12 h, whereas with other fungal biomass, nearly the same amount of H_2_ was produced, ranging between 5.86 and 6.30 mL/g DM by 24 h. After 24 h, the H_2_ production and conversion rate become equal resulting in stable cumulative H_2_ production. Although hay clover silage fermentation resulted in the same H_2_ generation as fungal biomass in 24 h (*P* > 0.05), it experienced a continuous gradual increase in H_2_ production resulting in the highest H_2_ amount of 9.02 mL/g DM after 48 h. Rapeseed meal produced the least total H_2_ (2.33 mL/g DM). It is reported that H_2_ is utilized to reduce CO_2_ to CH_4_ along with acetoclastic methanogenesis via VFAs fermentation due to hydrogenotrophic activity [61]. This could be explained by the initiation of CH_4_ production noticed at the beginning of 12 h when CO_2_ and H_2_ production rates followed a reducing trend (Fig. 4C). Digestion of fungal biomass produced in glucose and rapeseed meal generated the highest methane of about 14.56 mL/g DM and 12.57 mL/g DM, respectively. CH_4_ generation is strongly related to the stoichiometric ratio of VFA production; for example, the formation of propionic acid acts as an H_2_ sink, which reduces the production of CH_4_. Conversely, the production of acetate and butyrate is always accompanied by the production of H_2_ and CO_2_, which could induce the formation of CH_4_ [61].

From the experimental observation in this study, the fungal biomass, which had the highest gas production values, produced the highest VFAs ranging from 79.13 mmol/L to 99.23 mmol/L (Fig. 5A) in 48 h. Fungal biomass produce on glucose produced more acetic acid (up to 65.50 mmol/L) than hay clover silage (49.40 mmol/L) and rapeseed meal (49.40 mmol/L) in 48 h (*P* = 0.001). The acetic, propionic and butyric acids production rate increased until it almost doubled from 24 to 48 h for the fungal biomass. This increase reflects the stable H_2_ production and slower growth in methane noticed for fungal digestion assays after 24 h. Although the propionate concentration in silage increased from 19.57 mmol/L at 24 h to 28.17 mmol/L at 48 h, propionate rate of 0.056 mmol/L/h in 24 h, was reduced to 0.35 mmol/L/h by 48 h resulting in 22.2 mmol/L propionate content. In contrast, rapeseed meal exhibited a relatively constant propionate production rate of 0.043 mmol/L/h resulting in 20.6 mmol/L by the end of the digestion assay, while acetate and butyrate were at an almost similar formation rate as for hay clover silage. This would suggest that since the rapeseed meal resulted in a much lower amount of H_2_, a higher amount of propionate and a higher amount of CH_4_, the uptake of H_2_ for CH_4_ production was faster than for H_2_ production. Paula et al. [62] reported that the digestibility of rapeseed meal protein supplements may increase the CH_4_ formation and lower propionate proportion related to the digestibility of organic matter. Despite a reduction in the rate of propionate production for hay clover silage after 24 h, acetic acid was at a higher constant production rate, which was insufficient in enhancing the production of CH_4_.

According to Cone et al. [58], carbohydrate composition plays a vital role as highly soluble or readily rumen digestible carbohydrates can be expected to enhance the production of VFAs in the early hours, stimulating the digestibility of lower digestible carbohydrates. This increase in degradability seems to be a consequence of the stimulation of bacterial activity in the rumen. It can be concluded that the higher gas and VFA production from the fungal biomass may also attributed to the higher content of readly rumen digestible carbohydrates. Furthermore, the availability of highly digestible NDF in the feed may be directly proportional to the rumen's higher acetate/propionate (A/P) ratio [63]. This can also explain the A/P ratio of 2.6 and 3.5 in fungal biomass that was higher than hay clover silage (2.01 and 1.74) and rapeseed meal (2.26 and 1.84) at 24 h and 48 h, respectively (Fig. 5B). It is to be mentioned that propionate is an important VFA that significantly contributes to the glucose supply for the animal as a significant precursor for gluconeogenesis. In addition, higher VFAs, especially propionic acid, are essential and represent a positive relation with energy utilization efficiency [61, 64]. Therefore, higher VFA production, primarily acetic and propionic acid, throughout fungal biomass digestion could yield better energy in ruminants.

### Comparison of the methods

The IVDMD, and fermentation quality of the tested fungal biomass, silage and rapeseed meal ingredients, and the comparisons of the methods are presents in Table 3. When comparing between methods, it was observed that all methods gas, bag and TT significantly affected IVDMD of the five tested ingredients (*P* = 0.042) while the NH_4_^+^-N values were not affected by methods (*P* = 0.928). Surprisingly, despite the differences in time and pepsin addition between TT and gas methods, no significant difference in IVDMD values was observed (*P* = 0.232). The results from bag methods were much lower than TT method (*P* = 0.035). However, the bag methods demonstrated notably lower IVDM values compared to the TT methods (*P* = 0.035). Previous studies reported the connection between the reduction IVDMD and gas accumulation in in vitro and bag method [65]. Our results align with this statement, as the total gas generated in the bag method significantly differed from that in the gas method (*P* = 0.034). Digestibility measured in vitro using the Ankom bag is influenced by different sources of variation, type of sample, such quantity of sample, sample availability, and bag preparation (washing or non-washing) [44]. In this study, however, it is possible to consider that there might be limitations related to factors like the type and weight of the sample as well as the use of unwashed bags.

## Conclusion

The present study demonstrated the ruminal digestibility of the produced fungal biomass compared to silage and rapeseed meal based on their characteristics mainly CP and NDF. Although, significant difference was observed among the methods applied, the IVDMD, ammonia-N, gas and VFAs values were mainly influenced by the type of the ingredients (fungal biomass, hay clover silage and rapeseed meal) within each method. The higher IVDMD, gas production rate, and VFA generation observed in fungal biomass indicated its higher ruminal digestibility compared to the other feedstock used in this study in addition to its capacity to provide readily rumen degradable carbohydrates and CP, crucial for ruminal microorganisms. In particular, further studies should be designed to determine the potential of the fungal biomass as a supplement to the ruminant diets.

## Data Availability

All the data results involved in this study have been presented in the article.

## Abbreviations

AD: Anaerobic digestion
ADF: Acid detergent fiber
AIM: Alkali insoluble material
AO_Glu.: *A. oryzae* biomass grown on glucose
AO_VFAs FWCKM: *A. oryzae* biomass grown on VFAs effluents from food waste and chicken manure
AO_VFAs PPL: *A. oryzae* biomass grown on VFAs effluents from fermented potato protein liquor
Ca: Calcium
CF: Crude fiber
CH_4_: Methane
CO_2_: Carbon dioxide
CP: Crude protein
DM: Dry matter
Fe: Iron
FWCKM: VFAs effluents obtained from anaerobic digestion of food waste and chicken manure
H_2_: Hydrogen
IVDMD: In vitro dry matter digestibility
K: Potassium
Mg: Magnesium
Na: Sodium
NDF: Neutral detergent fiber
NH_4_^+^-N: Ammonium-N
OM: Organic matter
PPL: VFAs effluents obtained from anaerobic digestion of fermented potato protein liquor
tCOD: Total oxygen demand
TN: Total nitrogen
TVFAs: Total volatile fatty acids
VFAs: Volatile fatty acids
